# CXCR4 and TYROBP mediate the development of atrial fibrillation via inflammation

**DOI:** 10.1111/jcmm.17405

**Published:** 2022-05-23

**Authors:** Yan‐Fei Zhang, Ling‐Bing Meng, Meng‐Lei Hao, Xing‐Yu Li, Tong Zou

**Affiliations:** ^1^ Department of Cardiology, Beijing Hospital, National Center of Gerontology Institute of Geriatric Medicine, Chinese Academy of Medical Sciences Beijing China; ^2^ Neurology Department, Beijing Hospital, National Center of Gerontology Institute of Geriatric Medicine, Chinese Academy of Medical Sciences Beijing China; ^3^ Department of Geriatric Medicine Affiliated Hospital of Qinghai University Xining China; ^4^ School of Basic Medicine Peking University Beijing China

**Keywords:** arrhythmias, atrial fibrillation, CXCR4, inflammation, TYROBP

## Abstract

Atrial fibrillation (AF) is a rapid supraventricular arrhythmia. However, the pathogenesis of atrial fibrillation remains controversial. We obtained transcriptome expression profiles GSE41177, GSE115574 and GSE79768 from GEO database. WGCNA was performed, DEGs were screened, PPI network was constructed using STRING database. CTD database was used to identify the reference score of hub genes associated with cardiovascular diseases. Prediction of miRNAs of hub genes was performed by TargetScan. DIANA‐miRPath v3.0 was applied to make functional annotation of miRNA. The animal model of atrial fibrillation was constructed, RT‐PCR was used to verify the expression of hub genes. Immunofluorescence assay for THBS2 and VCAN was made to identify molecular. Design of BP neural network was made to explore the prediction relationship of CXCR4 and TYROBP on AF. The merged datasets contained 104 up‐regulated and 34 down‐regulated genes. GO and KEGG enrichment analysis results of DEGs showed they were mainly enriched in ‘regulation of release of sequestered calcium ion into cytosol’, ‘actin cytoskeleton organization’ and ‘focal adhesion’. The hub genes were CXCR4, SNAI2, S100A4, IGFBP3, CSNK2A1, CHGB, VCAN, APOE, C1QC and TYROBP, which were up‐regulated expression in the AF compared with control tissues. There was strong correlation among the CXCR4, TYROBP and AF based on the BP neural network. Through training, best training performance is 9.6474e‐05 at epoch 14, and the relativity was 0.99998. CXCR4 and TYROBP might be involved in the development of atrial fibrillation by affecting inflammation‐related signalling pathways and may serve as targets for early diagnosis and preventive treatment.

## 1 INTRODUCTION

Atrial fibrillation (AF) is a rapid supraventricular arrhythmia. It refers to uncoordinated atrial excitation and results in ineffective atrial contraction. AF is a serious cardiovascular condition that endangers human health in the 21st century, and its incidence increases with age.^1^, ^2^ Among people aged more than 80 years, the incidence of AF is as high as 7.5%,^3^ and the incidence of AF in men of all ages is higher than that in women.^1^ AF increases the risk of ischemic stroke and systemic arterial embolization; the risk of ischemic stroke in patients with AF is four‐ to five‐times that in non‐AF patients, leading to a mortality rate of nearly 20% and a disability rate of nearly 60%.^4^, ^5^ In addition, AF increases the risk of heart failure threefold and exacerbates the symptoms of heart failure.^4^, ^6^ Furthermore, patients with AF have a two‐fold increased risk of myocardial infarction.^7^ Finally, AF increases the risk of cognitive decline, dementia, Alzheimer's disease and vascular dementia,^8^ as well as increasing the risk of renal dysfunction.^9^ Recently, many researchers have applied bioinformatics analysis to identify differentially expressed genes (DEGs) in patients with AF, as well as the roles they play in different molecular functions, pathways and biological processes.^10^, ^11^

Bioinformatics technology has been widely used to find genetic changes in the processes of disease occurrence and development and can simultaneously analyse multiple changes in gene expression. It is a reliable means to find molecular diagnostic or therapeutic targets of disease. Wang and Wang used bioanalysis to screen multiple genes and molecules related to the occurrence and development of AF, which provided ideas for the early diagnosis and targeted therapy of AF.^12^, ^13^ Nielsen et al. conducted whole‐genome sequencing analysis and found multiple genetic risk sites related to the incidence of AF, suggesting that genetic factors may be involved in the incidence of AF, and that these genetic molecules may serve as early diagnosis and treatment targets for patients with AF complicated with stroke. DEGs were associated with the prognosis of patients with AF.^14^ Intensive studies should be performed to further explore the potential mechanisms involved in the abnormal expression of genetic molecular markers. Therefore, there is an urgent need to detect and analyse reliable gene targets linked with AF.

In the present study, we used bioinformatics technology to mine gene sequencing data, obtained from a public database, of patients with AF and compare these data with those of healthy individuals. We extracted high‐quality genetic datasets for further analysis. Functional enrichment analysis of the DEGs was performed using DAVID, Metascape and Gene Set Enrichment Analysis (GSEA). Weighted gene co‐expression network analysis (WGCNA) of the genes in the merged datasets was also performed. A summary of hub genes and the miRNAs that regulate these hub genes was obtained. Finally, we made a preliminary analysis of the role these DEGs and hub genes play in AF.

## 2 MATERIAL AND METHODS

### 2.1 Data from the GEO database

We obtained the transcriptome expression profiles GSE115574, GSE41177 and GSE79768 from the Gene Expression Omnibus (GEO) database (Table 1). The probes were transformed into homologous gene symbols via the platform's annotation information. The GSE115574 contained 28 AF and 31 sinus rhythm, GSE41177 dataset contained 32 AF and 6 sinus rhythm, while GSE79768 contained 14 AF and 12 sinus rhythm.

**TABLE 1:** Summary of atrial fibrillation microarray datasets from different GEO datasets

	Series	Platform	Affymetrix GeneChip	No. of Samples
1	GSE115574	GPL570	[HG‐U133_Plus_2] Affymetrix Human Genome U133 Plus 2.0 Array	59
2	GSE41177	GPL570	[HG‐U133_Plus_2] Affymetrix Human Genome U133 Plus 2.0 Array	38
3	GSE79768	GPL570	[HG‐U133_Plus_2] Affymetrix Human Genome U133 Plus 2.0 Array	26

### 2.2 WGCNA analysis

WGCNA (weighted gene co‐expression network analysis) is an analysis tool that can be used to describe correlation patterns among genes across microarray samples and find modules of highly correlated genes.^15^ In our study, the three GEO datasets were merged and normalized using Perl, and WGCNA analysis for merged datasets was conducted using the R package ‘WGCNA’.

### 2.3 Screening of DEGs

The DEGs were screened using the R package ‘limma’, and the cut‐off criterion was a *p*‐value < 0.05.

### 2.4 Functional annotation of DEGs

Kyoto Encyclopedia of Genes and Genomes (KEGG, http://www.genome.jp) analysis can provide specific pathways and link genomic information with higher‐order functional information. GO (Gene Ontology) analysis can annotate the functions of genes using terms from a dynamic, controlled vocabulary, which contains three aspects of biology: BP (biological processes), CC (cellular components) and MF (molecular functions). GSEA (Gene Set Enrichment Analysis) is a computational method that can execute GO and KEGG analysis for a given gene list. Metascape and DAVID are online analysis tools that provide a comprehensive gene list annotation and analysis resource. In our study, the GO and KEGG analyses of DEGs were performed using GSEA, Metascape and DAVID (*p* < 0.05).

### 2.5 Construction and analysis of the protein–protein interaction (PPI) network

STRING (http://string‐db.org) is an online database that can predict and provide a protein–protein interaction (PPI) network.^16^ Cytoscape software was used as the analysis tool; it can provide biological network analysis and two‐dimensional (2D) visualization for biologists. In our study, the PPI network was constructed using the STRING database and analysed by Cytoscape.

### 2.6 Identification of hub genes associated with cardiovascular diseases

The CTD database (comparative toxicogenomics database, http://ctdbase.org/) is a web‐based database that can identify relationships between genes/proteins and disease. In our study, the relationships between gene products and cardiovascular diseases were analysed using this database.^17^

### 2.7 Prediction of miRNAs of hub genes

TargetScan (www.targetscan.org) is an analysis tool that can perform predictive analyses and determine possible mechanisms for the co‐regulation of the expression of hundreds of genes expressed in different cell types. In our study, the miRNAs that regulated hub genes were screened for using TargetScan.

### 2.8 Functional annotation of miRNA

DIANA‐miRPath v3.0 (http://www.microrna.gr/miRPathv3) is an online software suite dedicated to conducting functional and pathway enrichment analyses for miRNAs. In our study, GO and KEGG pathway enrichment analyses were performed using miRPath (*p* < 0.05).

### 2.9 The construction of the animal model of atrial fibrillation

Twenty rats with a body weight of about 200 g were obtained from the Huafucang Biotechnology Co., Ltd. (Beijing, China), which were randomly divided into two groups, including control group (CON, *n* = 10) and the atrial fibrillation group (AF, *n* = 10). The rats in the AF group were narcotized with 4.5% isoflurane (0.8‐1 L/min). The rats were fixed in an upturned position and intubated in the external jugular vein or femoral vein. After that, electrocardiograph oscilloscope and electrocardiograph machine were connected to observe and record the II lead electrocardiograph, respectively. Arrhythmias may occur within 4–5 minutes after constant intravenous injection of aconitine solution at 1 pg/0.1 ml per minute with a micropump. The Animal Care and Use Committee of the Institute of Laboratory Animal Sciences, Chinese Academy of Medical Sciences and Peking Union Medical College (CAMS&PUMC) authorized the experimental ethics agreement.

### 2.10 RT‐PCR

Total RNA was extracted from the blood samples using the TRIzol® (Beijing Biolab Technology Co., Ltd.) and reverse transcribed into cDNA with the Servicebio®RT First Strand cDNA Synthesis kit (cat. no. G3330, Wuhan Servicebio Biotechnology Co., Ltd.) for 60 min at 42°C. Terminate the reaction by heating at 70°C for 5 min. RT‐qPCR was performed in a Light Cycler® 4800 System (Roche Diagnostics) with a specific set of primers for the amplification of secreted hub genes. Primers used were as follows in the Table 2. The thermocycling conditions used were as follows: 95°C for 15 s followed by 60°C for 60 s (a total of 30 cycles). The relative quantification units (relative quantification = 2^−ΔΔCt^, where Ct represents quantification cycle values) of each sample were calculated and presented as fold change of gene expression relative to the control group. GAPDH was used as an endogenous control.

**TABLE 2:** Primers and their sequences for RT‐PCR analysis

Primer	Sequence (5′–3′)
GAPDH‐hF	TGAAGGTCGGAGTGAACGGAT
GAPDH‐hR	CGTTCTCAGCCTTGACCGTG
CXCR4‐hF	TAAACACGAGGATGGCAAGA
CXCR4‐hR	AGGGCACTGAGACGCTGA
SNAI2‐hF	AGGGTATCATGGCACTTA
SNAI2‐hR	TTTACATCAGAATGGGTC
S100A4‐hF	ATGCCATTTCACCTCTAACT
S100A4‐hR	CTGTCTGCCATGCCAAGT
IGFBP3‐hF	CAGAGCACAGATACCCAGAA
IGFBP3‐hR	TAGCAGGTCAACAAGCATAG
CSNK2A1‐hF	GTGAGCCCTTGATGATTT
CSNK2A1‐hR	ACCCACGACCTCTTACCC
CHGB‐hF	GGGCAACAAGAGTAAGAC
CHGB‐hR	CTCTGCTTCCCAGGTTCT
VCAN‐hF	TGCCTTAATAATAGAGGG
VCAN‐hR	AGATAACGTGCAGTCAGT
APOE‐hF	CCAAAGTGCTGGGATTAGAGG
APOE‐hR	TCCAGTTCCGATTTGTAGGC
C1QC‐hF	ACGGAAGTCAGAGGAGGA
C1QC‐hR	CTGGAAGGAGCCGAATAG
TYROBP‐hF	TGCCTGAGCCTCCCGAGTA
TYROBP‐hR	CTGGGCGTGCATTCTTCA
PI3K‐hF	ATCCCGGAGTCGGAGCA
PI3K‐hR	CTGATTTGAGCTGATGCA
AKT‐hF	ATCCCCGGGGAAATTAG
AKT‐hR	AAGTTTTGATTTAGCCCC
TNF‐alpha‐hF	TGATGACCCGGGAACG
TNF‐alpha‐hR	ATGCGCTGCTAGATGCA

### 2.11 Immunofluorescence assay for THBS2 and VCAN

Washing three times with PBS (pH 7.4) (5 min/time), immersed the renal sections in EDTA antigen retrieval buffer (pH 8.0) (Servicebio G1206, Wuhan, China) to make antigen retrieval. Treat with PBS(PH7.4) (three times, 5 min/time), adding 3%BSA (Servicebio, G5001，Wuhan, China)to block non‐specific binding for 30 min. Throwing away the blocking solution, sections are incubated by CXCR4 antibody (dilution rate = 1:500, bs‐20317R, BIOSS, Beijing, China) (overnight, at 4°C). Washing the sections again with PBS (PH7.4), fluorescent secondary antibodies (diluteon rate = 1:5000) responding to the primary antibodies were added (room temperature, 50 min, dark condition). Then, incubated with DAPI solution (Servicebio, G1012, Wuhan, China) (room temperature, 10 min, darkness) to counterstaining nucleus.

Finally, using spontaneous fluorescence quenching reagent (5 min) (Servicebio, G1221, Wuhan, China) to make spontaneous fluorescence quenching and sealing the sections with anti‐fade mounting medium. The detecting process of TYROBP was the same as above using TYROBP antibody (dilution rate = 1:500, PAB23872, abnova). Fluorescence microscopy (Nikon NIKON ECLIPSE C1) showed that the nuclei were blue (excitation wavelength 330–380 nm and emission 420 nm) and the positive expression was red or green (FITC glows green by excitation wavelength 465–495 nm and emission 515–555 nm; CY3 glows red by excitation wavelength 510–560 nm and emission 590 nm).

### 2.12 Design of BP neural network

Before performing neural network modelling, the input and output of neural network need to be determined. In this experiment, due to the limitation of data, part of the input feature vectors in the disease risk determination model were reduced, and finally the two features data of CXCR4 and TYROBP, disease of AF were used as input vectors to conduct limited validation of the AF disease risk determination model, which can already reflect the health status of the body to a large extent due to the relatively large impact of these characteristics.

Corresponding to the three characteristics data entered is the individual's diagnostic result, and in the health status item, 1 represents that the individual is AF and 0 represents that the individual is normal. To fit the data and give the risk of AF, the model only needs a corresponding output.

### 2.13 The effect of CXCR4 and TYROBP on the myocardial cell via the PI3K/AKT signalling pathway

Normal myocardial cells and AF myocardial cells were purchased from Zhongke Quality Inspection Co., Ltd. The medium condition was DMEM (gibco, 12,800–017) + 10%FBS (Excell Bio, FSP500) + 1% double antibody (gibco, 15,140–122). The CXCR4 siRNA and TYROBP siRNA were compounded by Beijing Qingke biotechnology co., LTD. The expression level of CXCR4, TYROBP, PI3K, AKT and TNF‐alpha were detected after intervention of siRNA by RT‐qPCR.

### 2.14 Statistical analysis

The statistical analyses in this study were conducted using Perl, R software (version 3.5.3) and SPSS 20.0. Associations between hub genes and the status of samples were tested using Pearson's chi‐square test, while genes and their effects on AF based on univariate logistic proportional regression analysis were analysed. A receiver operating characteristic (ROC) curve was obtained using SPSS 20.0 and MedCalc software. Statistical analyses were conducted using SPSS software, version 24.0 (IBM Corp.), and MATLAB (R2014a, MathWorks.Inc). A *p*‐value < 0.05 was considered statistically significant.

## 3 RESULTS

### 3.1 Identification of DEGs

The GSE41177 dataset contained 9843 up‐regulated genes and 10 down‐regulated genes (Figure S1A). The GSE79768 dataset contained 6520 up‐regulated genes and 7603 down‐regulated genes (Figure S1B). The GSE115574 dataset contained 1316 up‐regulated genes and 1716 down‐regulated genes (Figure S1C). Finally, the merged datasets contained 104 up‐regulated genes and 34 down‐regulated genes (Figure S1D). Overlapping genes of the three datasets are shown in Figure S1E.

### 3.2 WGCNA analysis

In our study, WGCNA analysis was conducted using the R package ‘WGCNA’. A power value of 8 was the lowest power for which scale independence was below 0.8, and this was selected to produce a hierarchical clustering tree of 6301 genes (Figure S2A). The cluster of patients is shown in Figure S2B. In addition, a dendrogram and a heat map were used to quantify module similarity by correlation (Figure S2C). Interactions between these modules were then analysed (Figure S2D). The associations between clinical traits and the modules were identified based on the correlation between modules and clinical traits (Figure S2E).

### 3.3 Functional and pathway enrichment analysis of DEGs

The enrichment results of the GO and KEGG analyses of DEGs were obtained using DAVID; they were mainly enriched in ‘inflammatory response’, ‘inflammatory response to antigenic stimulus’, ‘actin cytoskeleton organization’, ‘focal adhesion’, ‘apical plasma membrane’, ‘cell surface’ and ‘PI3K‐Akt signaling pathway’ (Figure S3A–D).

The enrichment results of the GO and KEGG analyses of DEGs performed by Metascape were mainly enriched in ‘Staphylococcus aureus infection’, ‘inflammatory response’, ‘blood microparticle’, ‘endoplasmic reticulum lumen’ and ‘dimeric IgA immunoglobulin complex’ (Figure S3E–G).

The enrichment results of GO and KEGG analyses of DEGs, performed by GSEA, were mainly enriched in ‘cell volume homeostasis’, ‘cornified envelope’, ‘nuclear cyclin dependent protein kinase holoenzyme complex’, ‘copper ion homeostasis’, ‘regulation of heart morphogenesis’ and ‘myofibril assembly’ (Figure S4A–D; Tables 3 and 4).

**TABLE 3:** Gene ontology (GO) analysis by Gene set enrichment analysis (GSEA)

Term	Size	NES	*p*‐value	Rank at max	Leading edge
Up‐regulated					
GO_NUCLEAR_CYCLIN_DEPENDENT_PROTEIN_KINASE_HOLOENZYME_COMPLEX	15	1.691025	0.007737	796	tags = 20%, list = 3%, signal = 21%
GO_INORGANIC_CATION_TRANSMEMBRANE_TRANSPORTER_ACTIVITY	486	1.311503	0.034682	7158	tags = 38%, list = 30%, signal = 53%
GO_REGULATION_OF_POTASSIUM_ION_TRANSMEMBRANE_TRANSPORT	61	1.449976	0.03071	6514	tags = 43%, list = 28%, signal = 59%
GO_DELAYED_RECTIFIER_POTASSIUM_CHANNEL_ACTIVITY	36	1.402028	0.04466	2746	tags = 36%, list = 12%, signal = 41%
GO_CORNIFIED_ENVELOPE	117	1.360261	0.034749	8894	tags = 49%, list = 38%, signal = 78%
GO_CELL_VOLUME_HOMEOSTASIS	334	1.311257	0.046422	6299	tags = 36%, list = 27%, signal = 48%
Down‐regulated					
GO_REGULATION_OF_HEART_MORPHOGENESIS	27	−1.633	0.037549	2043	tags = 30%, list = 9%, signal = 32%
GO_COPPER_ION_HOMEOSTASIS	16	−1.53523	0.047244	4696	tags = 56%, list = 20%, signal = 70%
GO_MYOFIBRIL_ASSEMBLY	45	−1.37438	0.019588	2430	tags = 24%, list = 10%, signal = 27%
GO_ACTOMYOSIN_STRUCTURE_ORGANIZATION	72	−1.34398	0.049897	3311	tags = 33%, list = 14%, signal = 39%
GO_NEGATIVE_REGULATION_OF_SMOOTH_MUSCLE_CELL_MIGRATION	16	−1.76698	0.017544	2226	tags = 50%, list = 9%, signal = 55%
GO_NUCLEAR_LOCALIZATION_SEQUENCE_BINDING	20	−1.76468	0.010571	1339	tags = 45%, list = 6%, signal = 48%

**TABLE 4:** Kyoto Encyclopedia of genes and genomes (KEGG) analysis by gene set enrichment analysis (GSEA)

Term	Size	NES	*p*‐value	Rank at Max	Leading edge
Up‐regulated					
KEGG_METABOLISM_OF_XENOBIOTICS_BY_CYTOCHROME_P450	55	1.162694	0.02514735	5937	tags = 38%, list = 25%, signal = 51%
KEGG_CARDIAC_MUSCLE_CONTRACTION	72	1.0553582	0.037896827	4522	tags = 24%, list = 19%, signal = 29%
KEGG_ARRHYTHMOGENIC_RIGHT_VENTRICULAR_CARDIOMYOPATHY_ARVC	72	1.0394213	0.042209074	3636	tags = 19%, list = 15%, signal = 23%
KEGG_HISTIDINE_METABOLISM	28	1.5706778	0.008	5900	tags = 39%, list = 25%, signal = 52%
KEGG_TYROSINE_METABOLISM	42	1.5027974	0.028225806	6310	tags = 36%, list = 27%, signal = 49%
KEGG_REGULATION_OF_ACTIN_CYTOSKELETON	209	0.59139585	0.09861111	7522	tags = 25%, list = 32%, signal = 37%
Down‐regulated					
KEGG_VIRAL_MYOCARDITIS	65	−0.95915246	0.04722222	4654	tags = 42%, list = 20%, signal = 52%
KEGG_LYSOSOME	114	−0.95322895	0.05140562	2014	tags = 23%, list = 9%, signal = 25%
KEGG_PHOSPHATIDYLINOSITOL_SIGNALLING_SYSTEM	74	−0.9441891	0.04112036	2627	tags = 26%, list = 11%, signal = 29%
KEGG_GLUTATHIONE_METABOLISM	47	−0.88715667	0.0144814	3624	tags = 23%, list = 15%, signal = 28%
KEGG_MTOR_SIGNALLING_PATHWAY	81	−0.76322687	0.0463074	2159	tags = 19%, list = 9%, signal = 20%
KEGG_PHOSPHATIDYLINOSITOL_SIGNALLING_SYSTEM	88	−0.7046482	0.0354326	2159	tags = 17%, list = 9%, signal = 19%

In summary, through the above enrichment analysis, the DEGs related to the AF were mainly enriched in the biological processes of inflammation and infection. The results manifested that the AF might be correlated with the process of inflammation.

### 3.4 Construction and analysis of the PPI network

The PPI network of DEGs was constructed using the STRING online database and analysed with Cytoscape software (Figure S5A). Four different algorithms were employed to identify hub genes, and 10 hub genes were obtained (Figure S5B). A summary of the hub genes is shown in Table 5. The hub genes were CXCR4, SNAI2, S100A4, IGFBP3, CSNK2A1, CHGB, VCAN, APOE, C1QC and TYROBP (Figure S5C). The heat map of hub genes is shown in Figure S5D. Molecular Complex Detection (MCODE) analysis was employed to detect large PPI networks, which may represent molecular complexes, as shown in Figure S5E.

**TABLE 5:** Summary of hub genes

Symbol	Description	Function
SNAI2	Snail family transcriptional repressor 2	GO:0010957 negative regulation of vitamin D biosynthetic process
		GO:0070562 regulation of vitamin D receptor signalling pathway
		GO:0070563 negative regulation of vitamin D receptor signalling pathway
C1QC	Complement C1q C chain	GO:0030852 regulation of granulocyte differentiation
		GO:0030853 negative regulation of granulocyte differentiation
		GO:0045650 negative regulation of macrophage differentiation
APOE	Apolipoprotein E	GO:1905890 regulation of cellular response to very‐low‐density lipoprotein particle stimulus
		GO:1902995 positive regulation of phospholipid efflux
		GO:1903002 positive regulation of lipid transport across blood brain barrier
S100A4	S100 calcium binding protein A4	GO:0043122 regulation of I‐kappaB kinase/NF‐kappaB signalling
		GO:0043123 positive regulation of I‐kappaB kinase/NF‐kappaB signalling
		GO:0001837 epithelial to mesenchymal transition
TYROBP	TYRO protein tyrosine kinase binding protein	GO:0045081 negative regulation of interleukin‐10 biosynthetic process
		GO:0110090 positive regulation of hippocampal neuron apoptotic process
		GO:2001206 positive regulation of osteoclast development
VCAN	Versican	GO:0030205 dermatan sulfate metabolic process
		GO:0030207 chondroitin sulfate catabolic process
		GO:0030208 dermatan sulfate biosynthetic process
IGFBP3	Insulin‐like growth factor binding protein 3	GO:0045663 positive regulation of myoblast differentiation
		GO:0014912 negative regulation of smooth muscle cell migration
		GO:0043568 positive regulation of insulin‐like growth factor receptor signalling pathway
CSNK2A1	Casein kinase 2 alpha 1	GO:1903051 negative regulation of proteolysis involved in cellular protein catabolic process
		GO:0043154 negative regulation of cysteine‐type endopeptidase activity involved in apoptotic process
		GO:2000059 negative regulation of ubiquitin‐dependent protein catabolic process
CHGB	chromogranin B	GO:0036211 protein modification process
		GO:0006464 cellular protein modification process
		GO:0043687 post‐translational protein modification
CXCR4	C‐X‐C motif chemokine receptor 4	GO:0038160 CXCL12‐activated CXCR4 signalling pathway
		GO:0019064 fusion of virus membrane with host plasma membrane
		GO:0035470 positive regulation of vascular wound healing

### 3.5 Identification of hub genes

The CTD database showed that the hub genes targeted cardiovascular diseases, as shown in Figure S6.

### 3.6 Prediction and functional annotation of miRNAs associated with hub genes

The miRNAs that regulated the hub genes were screened for using TargetScan (Table 6). GO and KEGG analyses of miRNAs were performed using DIANA‐miRPath. GO and KEGG analyses showed significant enrichment in ‘axon guidance’, ‘NLS‐bearing protein import into nucleus’, ‘post‐translational protein modification’, ‘immune system process’, ‘stress‐activated MAPK cascade’, ‘MyD88‐dependent toll‐like receptor signaling pathway’, ‘cell death’ and ‘transcription, DNA‐templated’ (Figure S7).

**TABLE 6:** Summary of miRNAs that regulate the hub genes

	Gene	Predicted MiR
1	SNAI2	hsa‐miR‐203a‐3p.1 hsa‐miR‐429 hsa‐miR‐200b‐3p
2	C1QC	hsa‐miR‐4283 hsa‐miR‐4489 hsa‐miR‐185‐3p
3	APOE	hsa‐miR‐7704 hsa‐miR‐615‐5p hsa‐miR‐6742‐5p
4	S100A4	hsa‐miR‐6793‐3p hsa‐miR‐6859‐3p hsa‐miR‐325‐3p
5	TYROBP	hsa‐miR‐628‐5p hsa‐miR‐96‐3p hsa‐miR‐2681‐3p
6	VCAN	hsa‐miR‐203a‐3p.1 hsa‐miR‐124‐3p.1 hsa‐miR‐101‐3p.1
7	IGFBP3	hsa‐miR‐19a‐3p hsa‐miR‐19b‐3p hsa‐miR‐449b‐5p
8	CSNK2A1	hsa‐miR‐125a‐5p hsa‐miR‐125b‐5p hsa‐miR‐4319
9	CHGB	hsa‐miR‐371b‐3p hsa‐miR‐132‐3p hsa‐miR‐212‐3p
10	CXCR4	hsa‐miR‐140‐3p.1 hsa‐miR‐613 hsa‐miR‐1‐3p

### 3.7 Sensitivity and specificity of hub genes of the diagnosis for AF

The ROC curve of hub genes is shown in Figure S8. The associations between hub genes and the status of the sample are shown in Table 7. The genes and their effects on AF based on univariate logistic proportional regression analysis are shown in Table S1.

**TABLE 7:** Associations between hub genes and the status of the sample

	N	Pearson Correlation	Sig.(2‐tailed)		N	Pearson Correlation	Sig. (2‐tailed)
STATUS *SNAI2	61	0.508	<0.000	STATUS *VCAN	61	0.317	0.013
STATUS *C1QC	61	0.451	<0.000	STATUS *IGFBP3	61	0.357	0.005
STATUS *APOE	61	0.446	<0.000	STATUS *CSNK2A1	61	0.398	0.001
STATUS *S100A4	61	0.378	0.003	STATUS *CHGB	61	0.666	<0.000
STATUS *TYROBP	61	0.449	<0.000	STATUS *CXCR4	61	0.531	<0.000

Pearson's chi‐square test was used.

### 3.8 Results of RT‐qPCR for hub genes

As shown in Figure S9, the relative expression levels of CXCR4, SNAI2, S100A4, IGFBP3, CSNK2A1, CHGB, VCAN, APOE, C1QC and TYROBP were significantly higher in the AF group compared with the control group, according to the PCR results (*p* < 0.05, Figure S9).

### 3.9 The verification of protein expression of CXCR4 and TYROBP

By the immunofluorescence assay, protein expression of CXCR4 (Figure S10A) and TYROBP (Figure S10B) in the AF was higher than the control (*p* < 0.05).

### 3.10 Strong correlation among the CXCR4, TYROBP and AF based on the BP neural network

Through training, best training performance is 9.6474e‐05 at epoch 14 (Figure S11A), and the relativity was 0.99998 (Figure S11B). Then, the model verified the result, there were only no significant differences between predicted value and actual values (Figure S11C,D). Based on the above results, we could speculate that the level of CXCR4 and TYROBP expression might be predictive indexes for the AF.

### 3.11 CXCR4 and TYROBP might accelerate the inflammation to cause AF by activating the PI3K/AKT signalling pathway

Compared with the control myocardial cell, the expressions of CXCR4/PI3K/AKT/TNF‐alpha were higher in the AF myocardial cell (*p* < 0.05), and the inflammation response was strong. After down‐regulated the CXCR4 in the AF myocardial cell, the expressions of CXCR4/PI3K/AKT/TNF‐alpha were decreased significantly (*p* < 0.05), and the inflammation response was inhibited. (Figure S12A).

Compared with the control myocardial cell, the expressions of TYROBP /PI3K/AKT/TNF‐alpha were higher in the AF myocardial cell (*p* < 0.05), and the inflammation response was strong. After down‐regulated the TYROBP in the AF myocardial cell, the expressions of CXCR4/PI3K/AKT/TNF‐alpha were decreased significantly (*p* < 0.05), and the inflammation response was inhibited. (Figure S12B).

## 4 DISCUSSION

Atrial fibrillation is the most common arrhythmia.^18^, ^19^ Due to decreased cardiac output and auricular thrombosis, patients with AF are prone to related complications and increased risk of death.^20^, ^21^ Current treatments for AF mainly control symptoms and reduce the incidence of adverse events such as thrombosis, but long‐term use of anticoagulants will increase the risk of bleeding.^22^ However, the pathogenesis of AF remains controversial. Atrial enlargement, valvular disease and inflammation may all be involved in the occurrence and development of AF.^23^ Therefore, it is of great clinical significance to explore the mechanisms underlying AF and to find molecular targets for its early prevention, diagnosis and treatment.

Microarray technology allows the simultaneous analysis of changes in the expression of multiple genes to obtain gene sets that can predict conditions such as AF.^24^ Through comparative analysis of gene sequencing data from patients with AF and healthy individuals with sinus rhythm, we found that CXCR4, CHGB, IGFBP3, TYROBP, APOE, C1QC, SNAI2, CSNK2A1, S100A4 and VCAN were significantly more highly expressed in patients with AF. We believe that these hub genes are likely involved in the development of AF and may, therefore, serve as targets for the early diagnosis and prevention of AF. In particular, the role played by CXCR4 and TYROBP in the pathogenesis of AF deserves special attention.

Our result found that the CXCR4 and TYROBP might accelerate the inflammation to cause AF by activating the PI3K/AKT signalling pathway. Compared with the control myocardial cell, the expressions of CXCR4/TYROBP/PI3K/AKT/TNF‐alpha were higher in the AF myocardial cell (*p* < 0.05), and the inflammation response was strong. After down‐regulated the CXCR4 in the AF myocardial cell, the expressions of CXCR4/ TYROBP /PI3K/AKT/TNF‐alpha were decreased significantly (*p* < 0.05), and the inflammation response was inhibited. The PI3K family plays an important role in the transduction of intracellular signals and the pathogenesis of inflammation, obesity, and immune diseases.^25^ The PI3K/AKT signalling pathway plays different roles in normal physiological responses and inflammatory processes, including promoting cell proliferation, survival and differentiation. In atrial fibrillation, the PI3K/AKT pathway is overactivated.^26^ As one of the frequently activated signalling pathways in the occurrence of inflammation, the PI3K/Akt signalling pathway may be a target for the treatment of atrial fibrillation. Inflammation is the body's response to infection, tissue damage or cellular stress, and can restore tissue function through repair mechanisms. Inflammation can accelerate the development of atrial fibrillation.^27^ CXCR4 and TYROBP may activate the PI3K/AKT pathway by promoting the expression of inflammatory indicators, further leading to the development of atrial fibrillation.

CXCR4 (C‐X‐C motif chemokine receptor 4) is widely distributed and can be found in various locations within cells.^28^ Its molecular functions include actin binding, G protein‐coupled receptor activity, C‐C chemokine receptor activity, and ubiquitin protein ligase binding. Its biological functions include activation of MAPK activity, response to hypoxia, apoptotic processes, inflammatory responses, immune responses and positive regulation of cytosolic calcium ion concentration. The CXCR4‐related signalling pathway and molecular abnormalities may be involved in the development of many diseases.^29^, ^30^, ^31^ Xu et al. found that the CXCR4 receptor was involved in mediating the therapeutic effect of stromal cell‐derived factor‐1 in Parkinson's disease, suggesting that CXCR4 and its downstream pathway may be involved in the occurrence and development of Parkinson's disease and that CXCR4 could be used as a potential therapeutic target.^31^ Doring et al^29^ found that CXCR4 could regulate the occurrence and development of atherosclerosis by affecting the function of vascular endothelial cells. Furthermore, CXCR4 is involved in the occurrence and development of various diseases via the regulation of the inflammatory response. Mandal et al. found that CXCR4 could regulate the development and maturation of B cells by activating mitogen‐activated protein kinase extracellular signal regulating kinase; it then participated in affecting the immune function of the body and regulating the inflammatory response.^32^ In addition, CXCR4 could be involved in the development of atherosclerosis by regulating macrophage migration inhibitor (MIF) and macrophage formation, thereby affecting leukocyte recruitment and inflammatory responses, suggesting that CXCR4 may be a therapeutic target for atherosclerosis.^33^ Yu et al. found that CXCR4 could affect the prognosis of patients with colorectal cancer through downstream signalling molecule‐mediated inflammatory responses, again suggesting that it might be a potential therapeutic target.^34^ Furthermore, there is evidence to suggest that inflammation and related oxidative stress mediate the development of AF.^35^, ^36^ Zou et al. performed a bioinformatics analysis and found multiple molecular markers involved in AF with stroke; they believed that CXCR4 was also involved in the occurrence of stroke in patients with AF.^11^ Through our bioanalysis, we found that CXCR4 expression was high in patients with AF and low in adults with sinus rhythm. We speculated that CXCR4 was involved in the occurrence and development of AF via the regulation of inflammatory responses and immune responses, as well as positive regulation of cytosolic calcium ion concentration and other mechanisms. CXCR4 may be a target for the early diagnosis and prevention of AF, so the relevant mechanisms are worth further exploration.

The transmembrane immune signalling adaptor, TYROBP, is mainly located at the plasma membrane, cell surface and integral components of the membrane.^37^ It influences signalling receptor binding, protein binding, microglial cell activation involved in immune responses, signal transduction, positive regulation of gene expression, positive regulation of natural killer cell activation, positive regulation of macrophage fusion, intracellular signal transduction, positive regulation of tumour necrosis factor biosynthetic processes and the apoptotic signalling pathway. TYROBP‐related signal pathways and molecular abnormalities can also cause a variety of abnormal physiological states in the body. Through analysing sequence data of patients with Alzheimer's disease, Pottier et al. found that the abnormal expression of TYROBP is involved in the occurrence and development of Alzheimer's disease and may be useful as an early diagnosis and treatment target.^38^ Using bioinformatics, Pan et al. found several molecules that were abnormally expressed in patients with osteosarcoma, among whom TYROBP was highly expressed, suggesting that it might be a potential therapeutic target.^39^ Furthermore, there is evidence that TYROBP may be involved in the occurrence and development of a variety of diseases, through the regulation of the body's inflammatory response. Yin and colleagues suggested that TYROBP may be involved in the development of Alzheimer's disease by affecting microglial cell activity and the formation of related inflammatory plaques.^40^ Ashton et al. identified multiple abnormally expressed genes by performing single‐cell gene expression analysis of the peripheral blood of patients with rheumatoid arthritis, systemic lupus erythematosus or diabetes and proposed that TYROBP may be involved in the occurrence and development of diabetes by regulating immune function and inflammation.^41^ There is evidence that inflammation and associated oxidative stress are involved in the onset of AF. It has been shown that patients with AF had elevated levels of serum inflammatory biomarkers and increased expression of inflammatory markers in atrial tissue, which may promote and maintain AF.^35^ Korantzopoulos et al. suggested that reactive oxygen species (ROS) in the atrium could affect atrial remodelling and participate in the occurrence and development of AF.^42^ Therefore, the study of inflammation and oxidative stress as diagnostic and therapeutic targets for patients with AF shows great potential. We found through our bioanalysis that the expression of TYROB was high in patients with AF and low in adults with sinus rhythm. We hypothesized that CXCR4, by adjusting the inflammatory response, signal transduction, positive regulation of gene expression, positive regulation of macrophage fusion and intracellular signal transduction, could be linked to a variety of mechanisms involved in the occurrence of AF. TYROB may be a suitable target for the early diagnosis, early prevention and treatment of AF. The relevant mechanism is worthy of in‐depth study and provides new avenues for studying the pathogenesis of AF.

Despite the rigorous bioinformatics analysis conducted in this study, there are still some shortcomings. This study lacks an in‐depth verification of the proposed mechanisms. The reliability of our results could be improved by animal experiments and comprehensive verification using clinical samples.

In conclusion, bioanalysis can effectively identify differentially expressed genes between patients with atrial fibrillation and healthy individuals with sinus rhythm. Specifically, CXCR4 and TYROBP mediate the development of AF through inflammation. These differentially expressed genes may be involved in the development of AF by affecting inflammation‐related signalling pathways and may serve as targets for early diagnosis and preventive treatment. Our study provides new evidence and ideas for the further exploration of the pathogenesis and treatment of atrial fibrillation.

## AUTHOR CONTRIBUTIONS

**Yan‐fei Zhang:** Conceptualization (equal); project administration (equal); writing – review and editing (equal). **Ling‐bing Meng:** Formal analysis (equal); methodology (equal); visualization (equal). **Meng‐lei Hao:** Methodology (equal); writing – original draft (equal). **Xing‐yu Li:** Formal analysis (equal); methodology (equal); visualization (equal); writing – original draft (equal). **Tong Zou:** Data curation (equal); methodology (equal); writing – original draft (equal).

## CONFLICT OF INTEREST

The authors declare that they have no competing interests.

## Supporting information


Appendix S1

## Data Availability

The datasets used and/or analyzed during the current study are available from the corresponding author on reasonable request.
