# Supplementary material for: CXCR4 and TYROBP mediate the development of atrial fibrillation via inflammation
Source: J Cell Mol Med. 2022 May 23;26(12):3557–67. doi: 10.1111/jcmm.17405 (PMC9189330; doi:10.1111/jcmm.17405)
Supplement: Supplementary file 1 — Appendix S1 [file JCMM-26-3557-s001.docx]

| Table S1. Genes and their effects on atrial fibrillation based on univariate logistic proportional regression analysis. | | | | |
| --- | --- | --- | --- | --- |
| GENE | OR | 95% CI | P-value |  |
| SNAI2 | 4.515 | 1.950-10.456 | .000 |  |
| C1QC | 3.744 | 1.650-8.495 | .002 |  |
| APOE | 4.997 | 1.853-13.480 | .001 |  |
| S100A4 | 2.982 | 1.332-6.677 | .008 |  |
| TYROBP | 3.213 | 1.549-6.666 | .002 |  |
| VCAN | 2.069 | 1.134-3.775 | .018 |  |
| IGFBP3 | 2.511 | 1.258-5.012 | .009 |  |
| CSNK2A1 | 3.276 | 1.469-7.303 | .004 |  |
| CHGB | 24.387 | 4.596-129.406 | .000 |  |
| CXCR4 | 6.207 | 2.297-16.774 | .000 |  |

**Figure legends:**

**
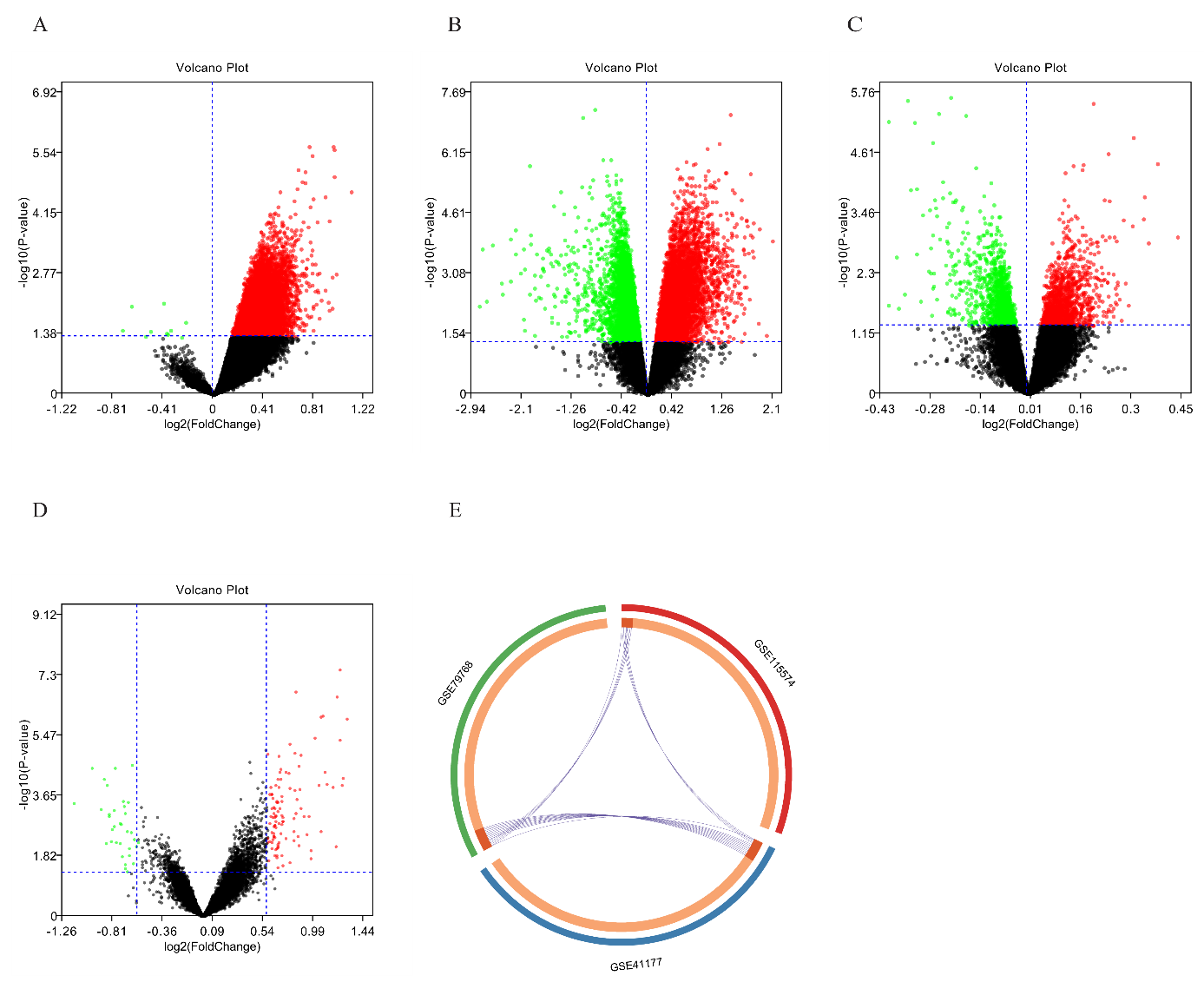
**

Figure. S1 The genes are differentially expressed genes (DEGs) between the atrial fibrillation group and the sinus rhythm group. (A) Volcano plots of DEGs in GSE41177. (B) Volcano plots of DEGs in GSE79768. (C) Volcano plots of DEGs in GSE115574. (D) Volcano plots of DEGs in the merged datasets. (E) Overlapping DEGs in the three datasets.


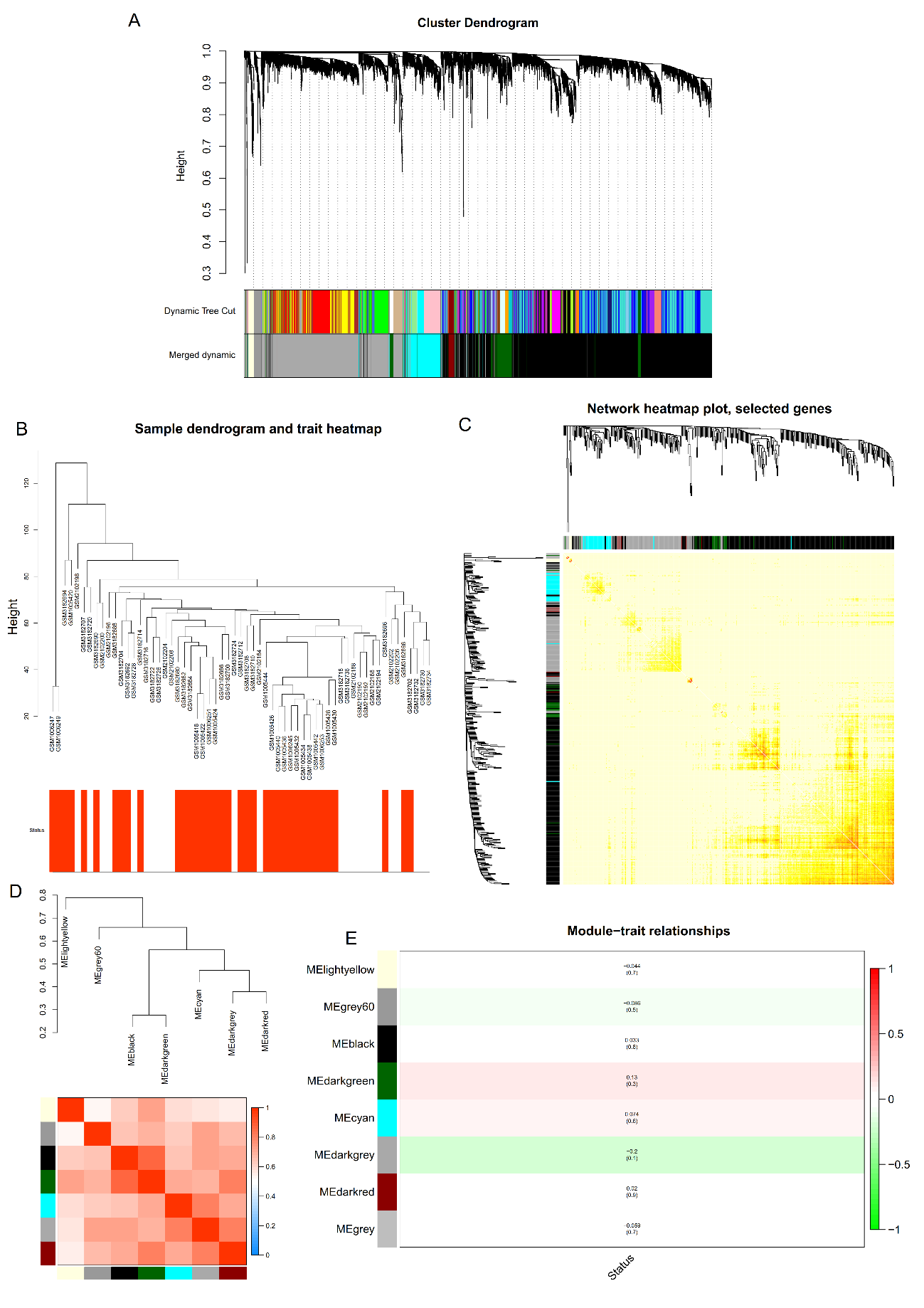


Figure. S2 WGCNA (weighted gene co-expression network analysis) of the genes in the merged datasets. (A) Repeated hierarchical clustering tree of the 6,301 genes. (B) A cluster of patients with clinical information; the red line represents patients with atrial fibrillation (C) A dendrogram and a heat map of genes. (D) Interactions between these modules. (E) Associations between clinical traits and the modules.


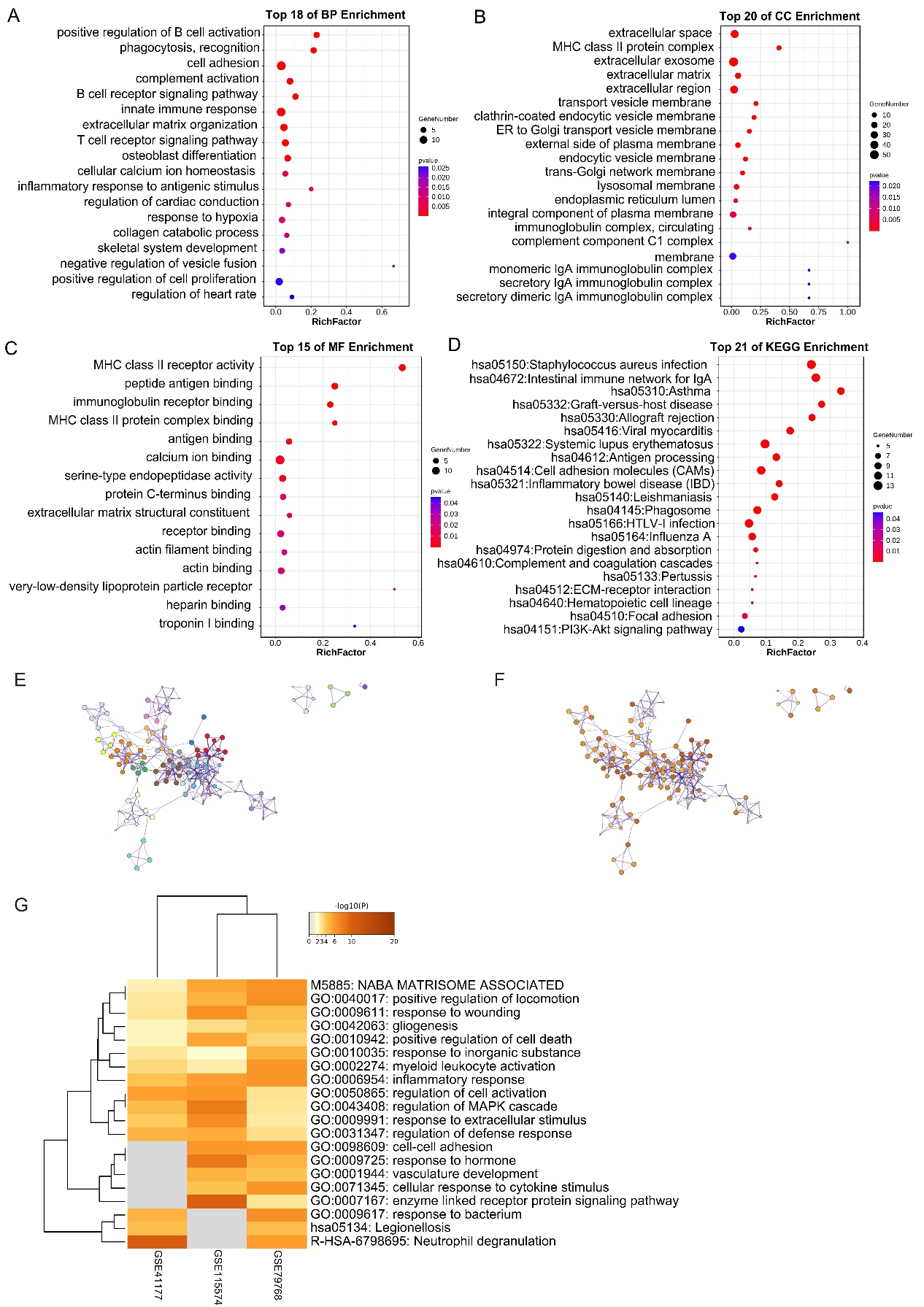


Figure. S3 Gene functional enrichment analysis of the DEGs by DAVID and Metascape. (A) Gene ontology (GO) analyses according to biological processes (BP) by DAVID. (B) GO analyses according to cellular components (CC) by DAVID. (C) GO analyses according to molecular functions (MF) by DAVID. (D) Kyoto Encyclopedia of Genes and Genomes (KEGG) analyses of the DEGs by DAVID. (E-G) GO analyses and KEGG analyses by Metascape.


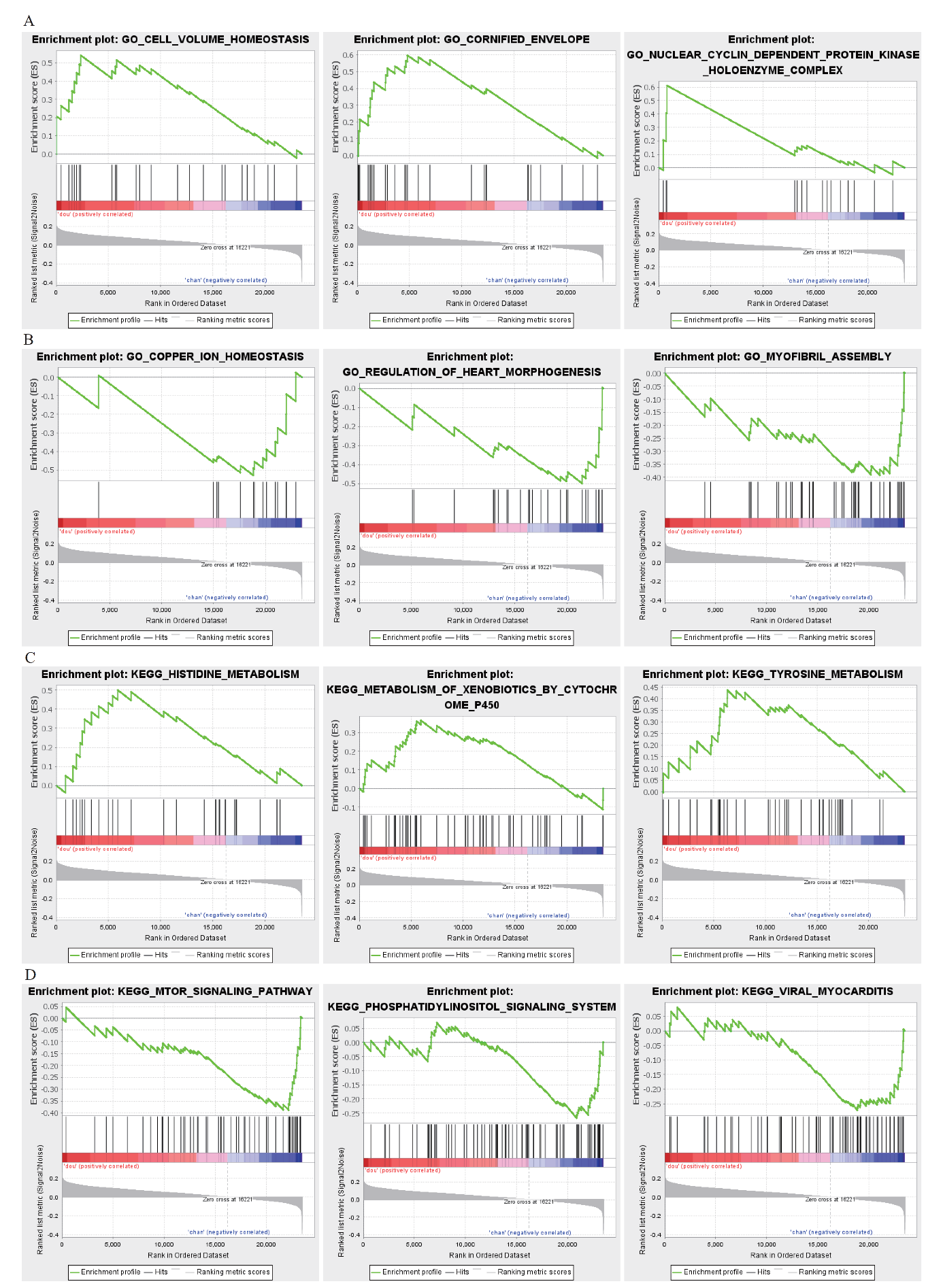


Figure. S4 Gene functional enrichment analysis of the DEGs by GSEA. (A) GO analyses of the highly expressed DEGs. (B) GO analyses of the low expressed DEGs. (C) KEGG analyses of the highly expressed DEGs. (D) KEGG analyses of the low expressed DEGs.


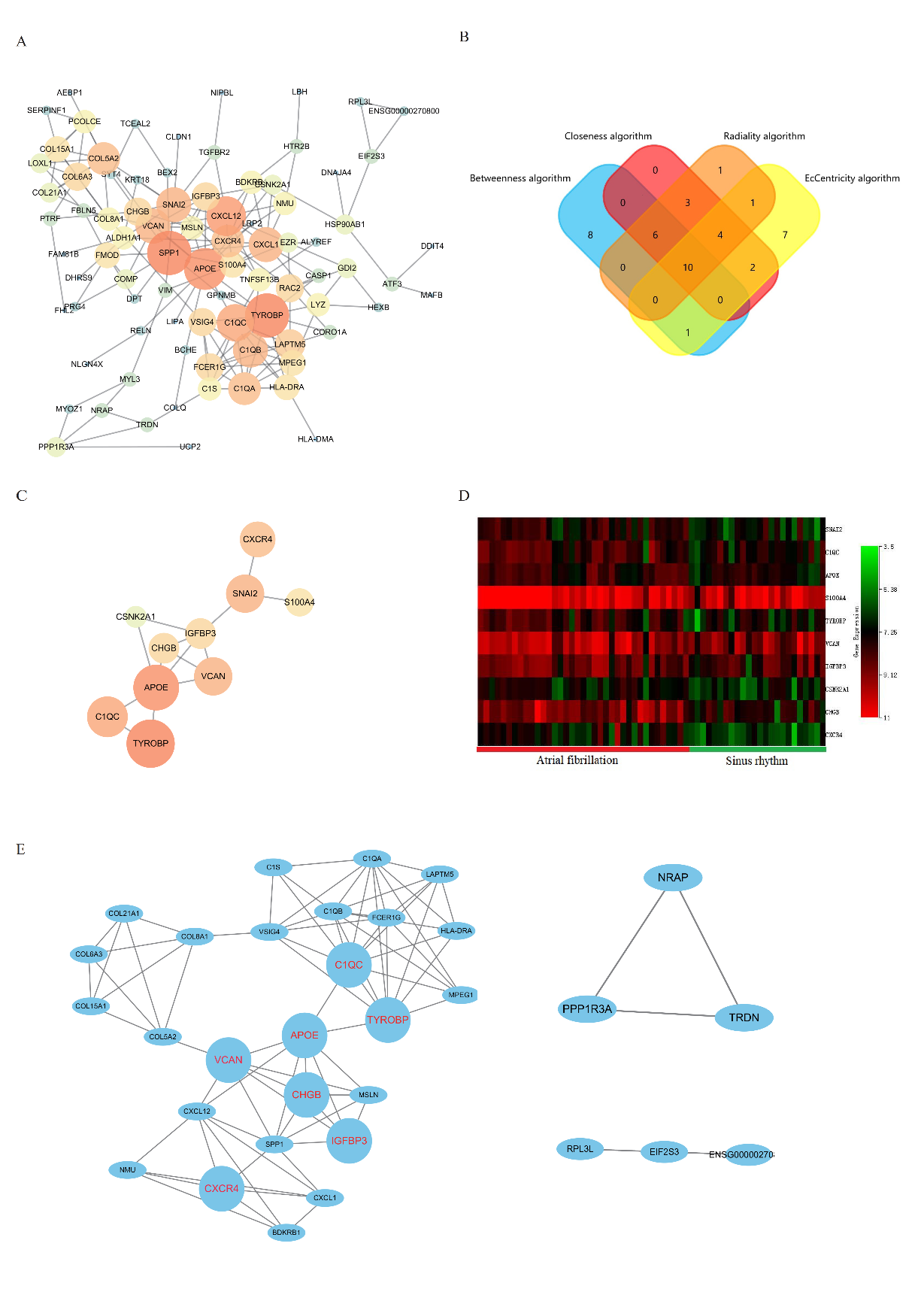


Figure. S5 Relationships between DEGs. (A) Protein–protein interaction (PPI) network, the larger the number of connections, the larger the protein. (B) The common hub genes identified using different algorithms. (C) The common hub genes of the PPI network. (D) Heat maps of the common hub genes. (E) MCODE analysis of the common hub genes.


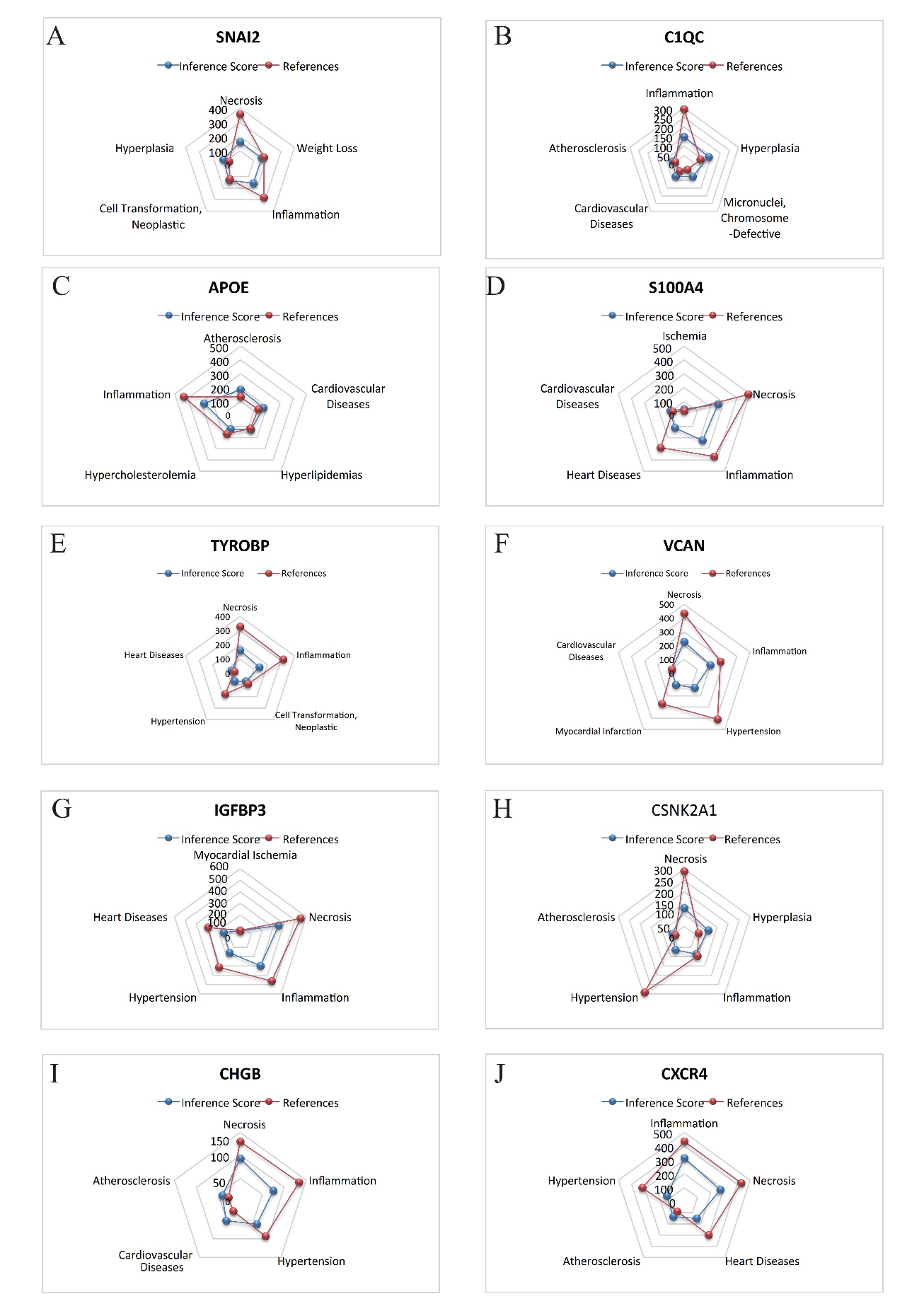


Figure. S6 Relationships of the atrial fibrillation group and sinus rhythm group related to DEGs, based on the CTD database. (A) SNAI2 (B) C1QC (C) APOE (D) S100A4 (E) TYROBP (F) VCAN (G) IGFBP3 (H) CSNK2A1 (I) CHGB (J) CXCR4.


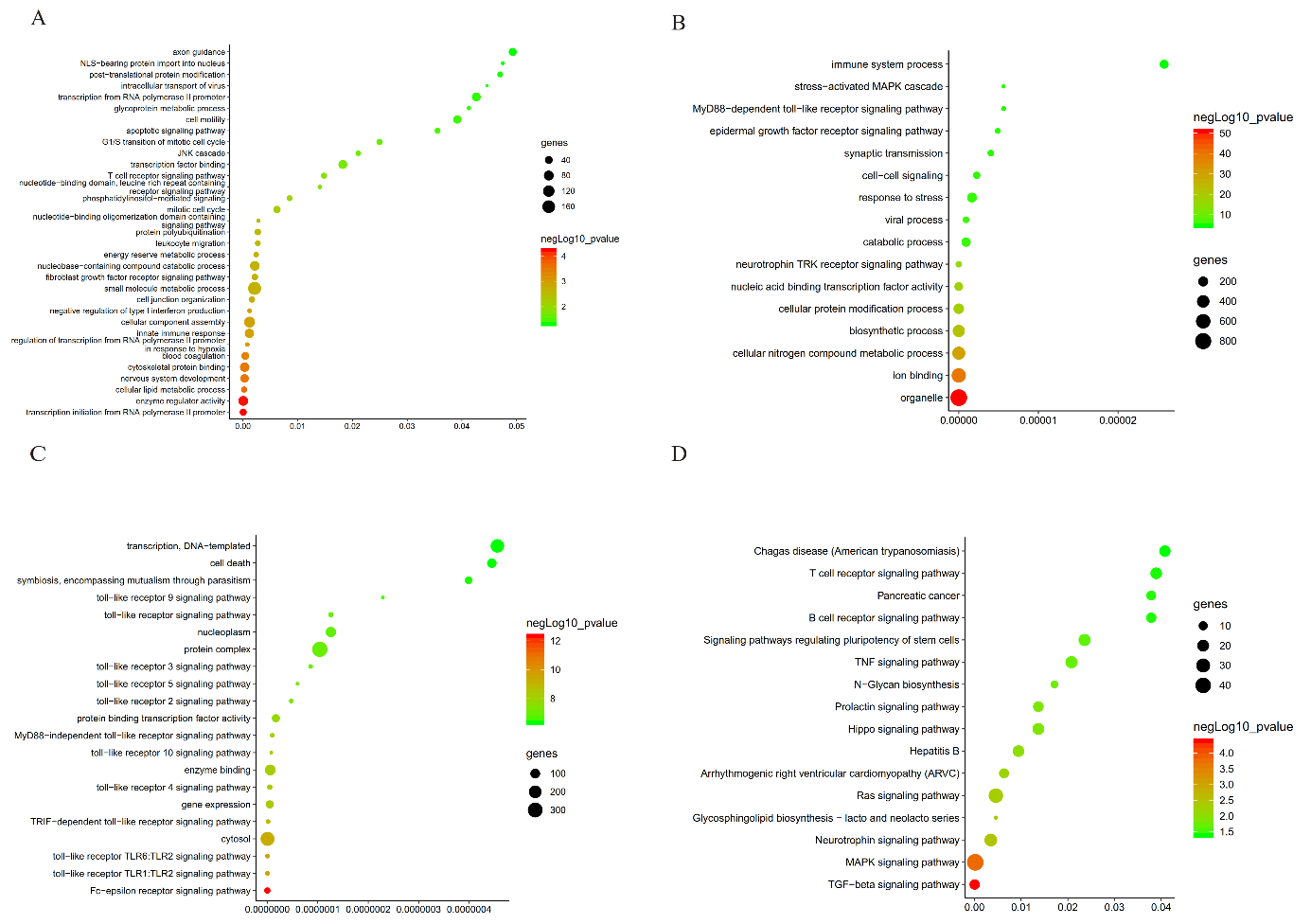


Figure. S7 Functional and pathway enrichment analysis of miRNAs which could regulate hub genes. (A) BP analyses (B) CC analyses. (C) MF analyses. (D) KEGG analyses of the miRNAs.


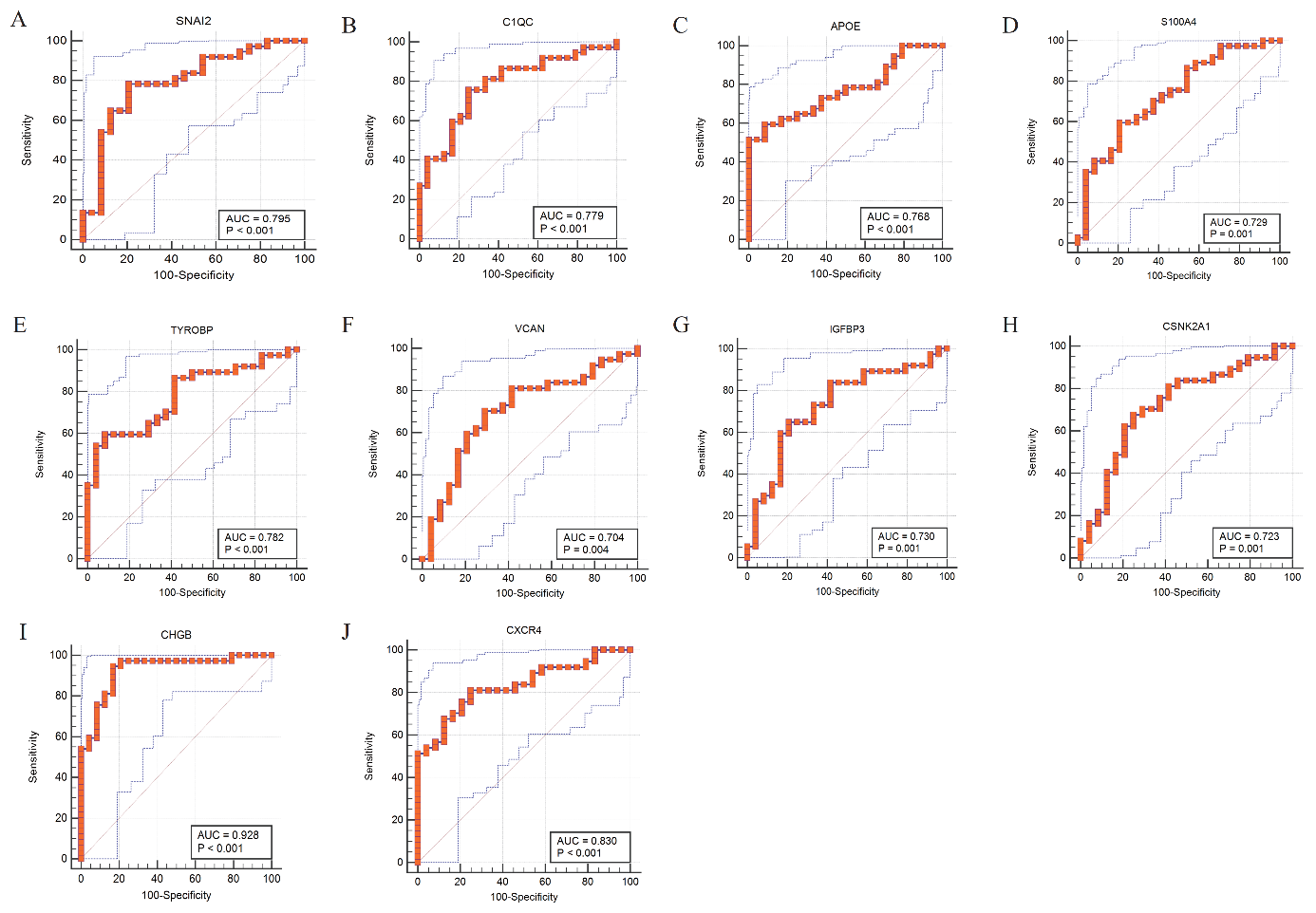


Figure. S8 Receiver operating characteristic (ROC) curve of hub genes. (A) SNAI2 (B) C1QC (C) APOE (D) S100A4 (E) TYROBP (F) VCAN (G) IGFBP3 (H) CSNK2A1 (I) CHGB (J) CXCR4.


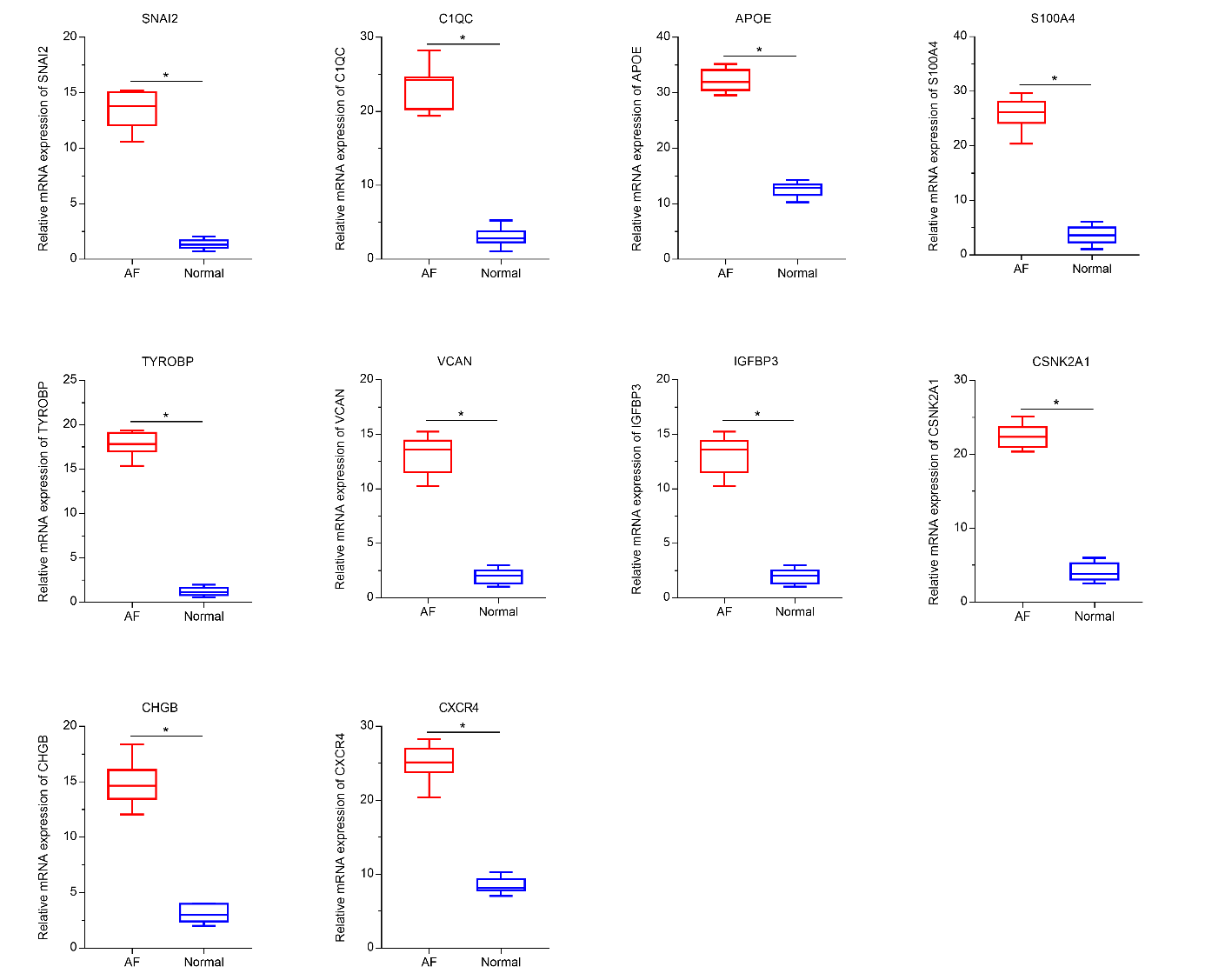


Figure. S9 Results of RT-qPCR for CXCR4, SNAI2, S100A4, IGFBP3, CSNK2A1, CHGB, VCAN, APOE, C1QC, and TYROBP. They were significantly higher in the AF group compared with the control group.


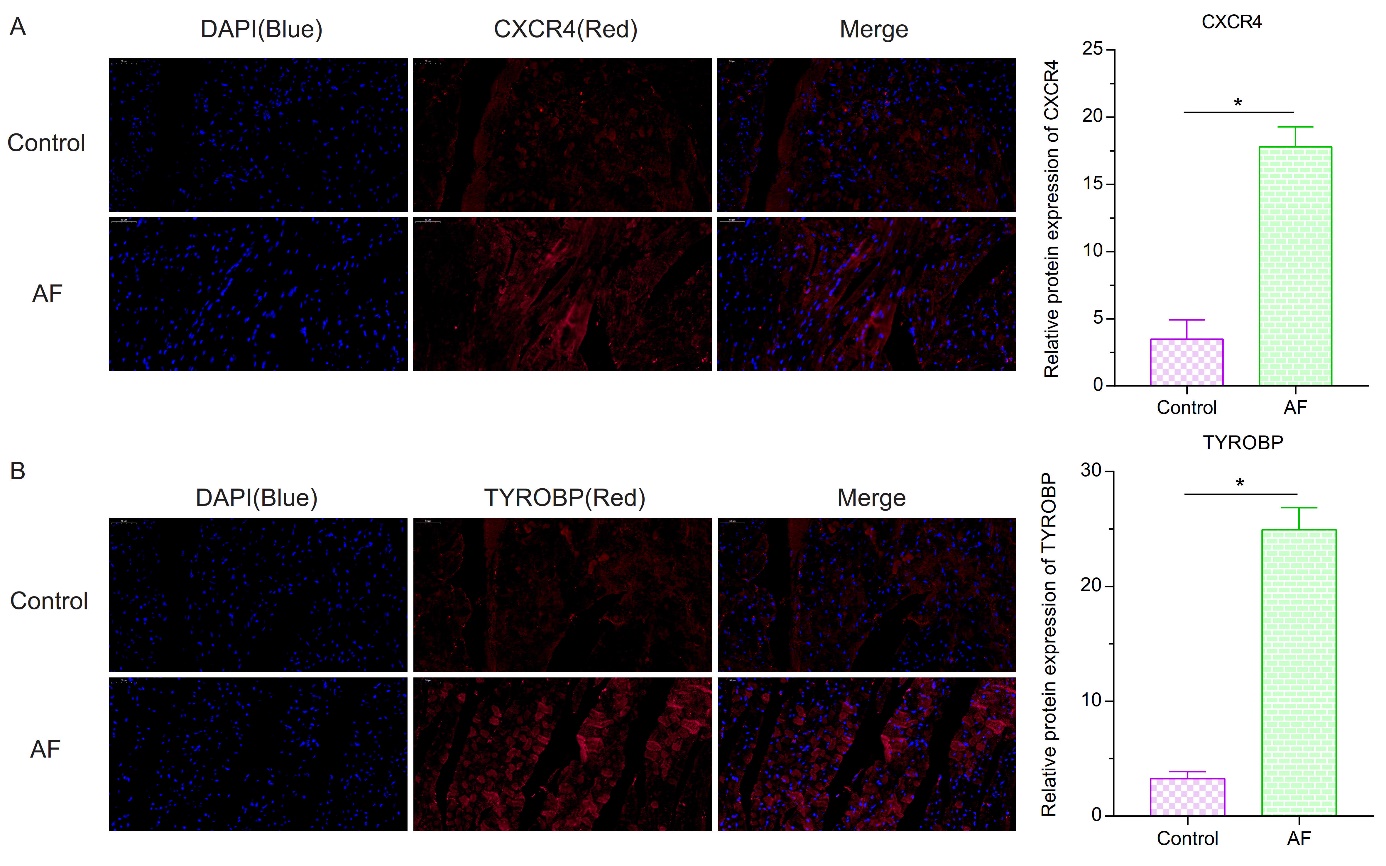


Figure. S10 The verification of protein expression of CXCR4 and TYROBP by the immunofluorescence assay. Protein expression of (A) CXCR4 and (B) TYROBP in the AF was higher than the control.


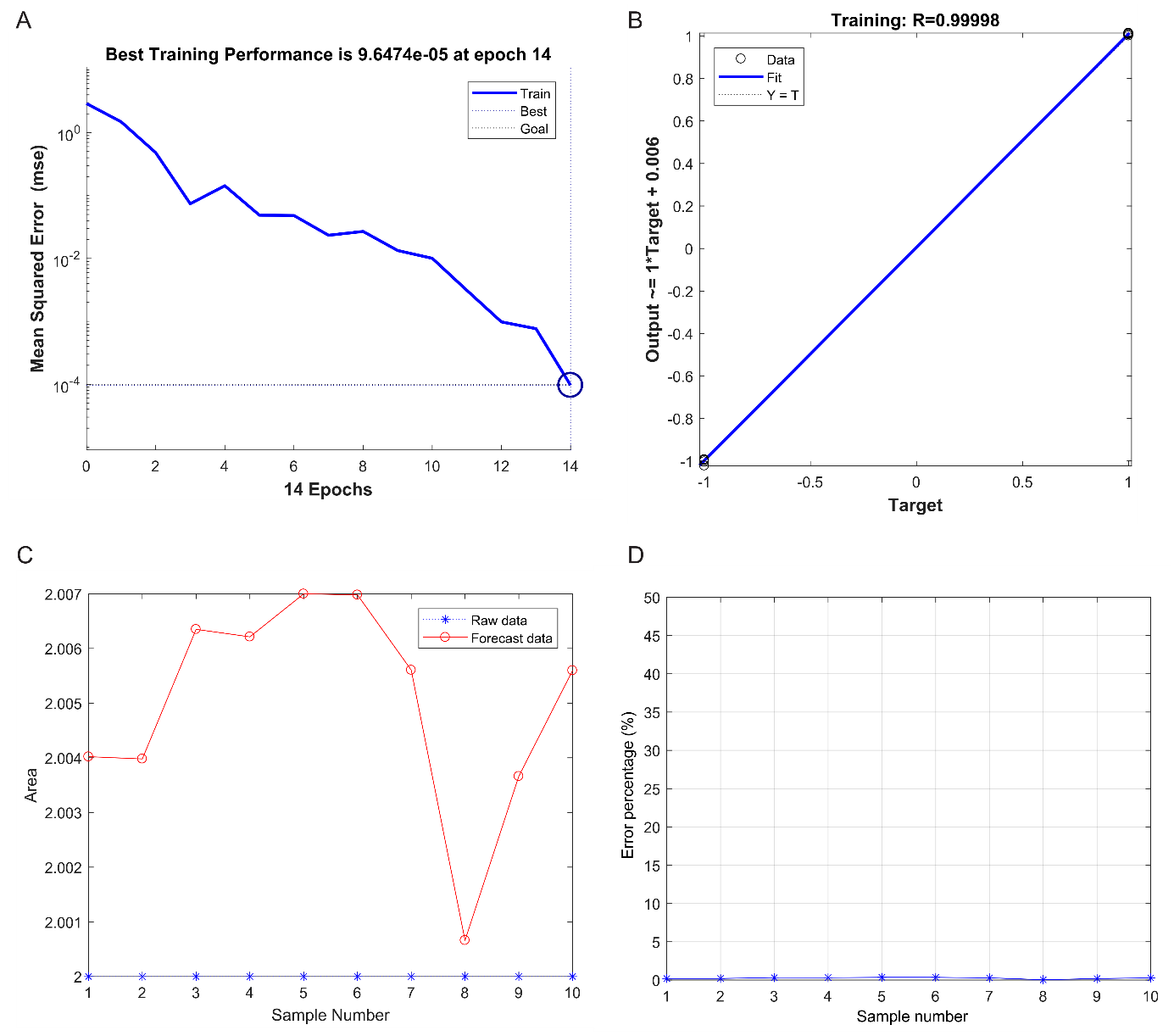


Figure. S11 Strong correlation among the CXCR4, TYROBP and AF based on the BP neural network.


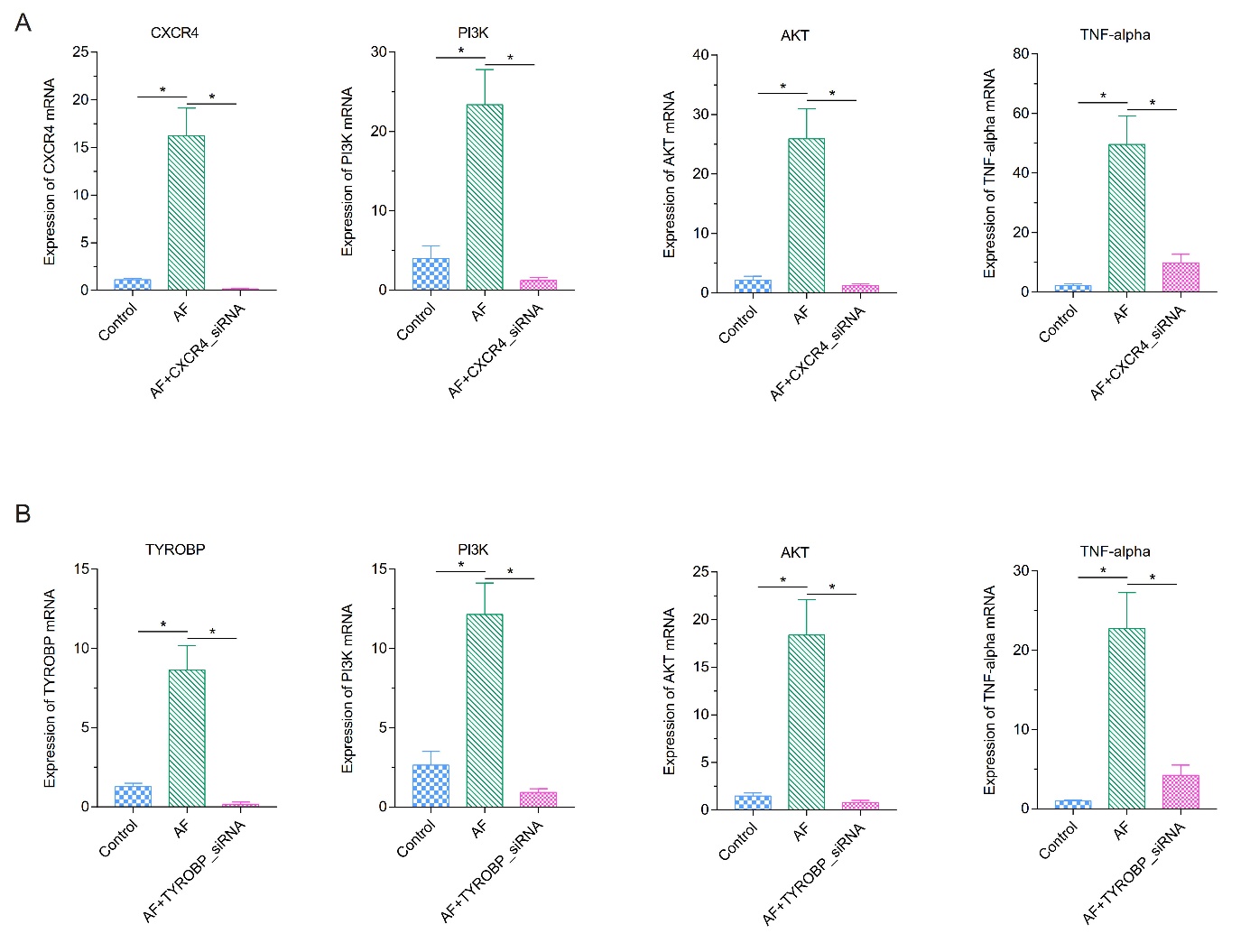


Figure S12. CXCR4 and TYROBP might accelerate the inflammation to cause AF by activating the PI3K/AKT signaling pathway. (A) Effect of CXCR4 on the myocardial cell via the PI3K/AKT signaling pathway. (B) Effect of TYROBP on the myocardial cell via the PI3K/AKT signaling pathway.
